# An Overlapping-Signal Separation Algorithm Based on a Self-Attention Neural Network for Space-Based ADS-B

**DOI:** 10.3390/s26041351

**Published:** 2026-02-20

**Authors:** Ziwei Liu, Shuyi Tang, Yehua Cao, Shanshan Zhao, Leiyao Liao, Gengxin Zhang

**Affiliations:** School of Communications and Information Engineering, Nanjing University of Posts and Telecommunications, Nanjing 210003, China; lzw@njupt.edu.cn (Z.L.); 1024010322@njupt.edu.cn (S.T.); 1222014804@njupt.edu.cn (Y.C.); 20230117@njupt.edu.cn (L.L.); zgx@njupt.edu.cn (G.Z.)

**Keywords:** ADS-B, signal separation, self-attention, satellite communications, blind source separation

## Abstract

Space-based automatic dependent surveillance–broadcast (ADS-B) systems offer the potential for comprehensive global aircraft surveillance. However, they face substantial challenges due to severe signal collisions resulting from the simultaneous reception of asynchronous ADS-B transmissions from multiple aircraft within a satellite’s expansive coverage area. Traditional collision mitigation approaches, such as serial interference cancellation and multichannel blind source separation, often have high computational costs, impose strict signal structure constraints, or rely on multiple-antenna configurations, all of which limit their practicality in satellite scenarios. To address these limitations, this paper proposes two novel deep learning–based models, designated SplitNet-2 and SplitNet-3. SplitNet-2 leverages a Transformer-inspired self-attention architecture specifically designed to separate two overlapping ADS-B signals, while SplitNet-3 employs a convolutional residual U-shaped network optimized for disentangling three simultaneous, colliding signals. Extensive simulations under realistic satellite reception conditions demonstrate that the proposed models significantly outperform conventional methods, achieving lower bit error rates (BERs) and improved demodulation accuracy. These advancements offer a promising solution to the critical problem of underdetermined signal separation in space-based ADS-B reception and significantly enhance the reliability and coverage of satellite-based ADS-B surveillance systems.

## 1. Introduction

Automatic dependent surveillance–broadcast (ADS-B) is a next-generation air traffic surveillance technology proposed by the International Civil Aviation Organization (ICAO) to advance global air traffic control modernization [1]. Unlike traditional primary radar, which relies on ground echoes, ADS-B system uses an onboard transmitter to autonomously broadcast an aircraft’s identity, position, altitude, and velocity. These messages are received in real time by ground or satellite receivers to provide continuous surveillance of the aircraft [2]. ADS-B system has substantially improved the accuracy and coverage of air traffic surveillance and is regarded as a key enabler of future aviation safety and efficiency.

However, current ground-based ADS-B systems rely primarily on terrestrial base stations to provide airspace surveillance. This architecture achieves limited coverage per station and has high deployment costs, and it is difficult to deploy over oceans, rainforests, and other complex terrain. As a result, such systems are used mainly in airspace with dense activity. To overcome these limitations, space-based ADS-B has emerged. A space-based ADS-B system sends information to low earth orbit (LEO) satellites equipped with ADS-B receivers, and the LEO satellites then transmit these messages to ground base stations. It has the advantages of wide coverage and low deployment cost, and it can also manage airspace traffic and improve airspace management efficiency.

Despite the significant advantages of space-based ADS-B, it still faces the serious challenge of message collisions when receiving signals globally. In a typical space-based ADS-B system, each aircraft transmits ADS-B messages using the 1090 MHz Extended Squitter (1090ES) format [3]. When multiple aircraft transmit simultaneously within the same airspace, the receiver observes overlapping messages. Unlike ground stations, whose coverage is severely limited, LEO satellites can cover regions that span thousands of kilometers. They can also receive a large volume of signals concurrently, which makes signal collisions exceedingly common. As shown in [4], when the fleet size reaches 3000 aircraft, nearly 90% of messages overlap. Without effective mitigation, decoding success rates decline sharply, thereby delaying the ingestion of flight information into Air Traffic Control (ATC) systems and jeopardizing flight safety. Incidents such as the disappearance of MH370 further underscore the urgent need for continuous global surveillance [5]. Therefore, research on collision separation for space-based ADS-B signals is of considerable significance [6,7,8].

To date, extensive research has been conducted worldwide to address ADS-B signal collisions. In general, existing techniques can be classified into two groups: multi-antenna array schemes and single-antenna reception schemes. The former exploits the spatial dimension provided by antenna arrays to disentangle overlapping signals according to their differing angles of arrival. For example, ref. [9] proposed the Projection Algorithm (PA) and the Extended Projection Algorithm (EPA). By performing orthogonal projection separation on signals received by multiple antennas, they achieved demixing of secondary radar reply signals. Such array-based algorithms can separate multiple signals simultaneously, but when the differences in the angles of arrival of overlapping signals are smaller than the antenna array’s angular resolution, spatial filtering fails [10]. Ref. [11] formulated ADS-B signal separation as an optimization problem and tackled the resulting nonconvex blind adaptive beamforming task with the alternating direction method of multipliers (ADMM). However, in many practical scenarios, such as space-based ADS-B, constraints on satellite size and cost restrict receivers to a single antenna and make large arrays infeasible [12]. Consequently, research has increasingly shifted toward collision signal separation algorithms designed for single-antenna operation. Because a single-antenna ADS-B receiver lacks spatial diversity, signal separation must rely solely on intrinsic differences in power, frequency, or timing [13]. For example, Yu et al. proposed a reconstruction and cancellation algorithm that first demodulates the stronger signal and then subtracts it from the composite to retrieve the weaker one [14]. However, techniques based on power can typically separate only two signals, and their performance degrades sharply when the power disparity is small [15]. Galati et al. introduced the Projection Algorithm for a Single Antenna (PASA) for Mode S decoding. The technique reconstructs the composite signal captured by a single antenna into multichannel data and then applies an orthogonal projection algorithm to separate the individual signals [16].

Recent studies have also begun applying deep learning to separate colliding ADS-B signals [17]. Because the task resembles single-channel speech source separation, researchers have ported popular speech separation network architectures to the ADS-B domain and employed end-to-end models to process overlapping messages. Yan et al. constructed a semi-physical ADS-B dataset by augmenting real captured signals with controlled variations in signal-to-noise ratio (SNR), carrier frequency offset, and relative delay to train deep learning models. Using a multiscale convolutional TasNet that fuses temporal features at different scales for mask estimation, their system achieved a measured decoding accuracy of 90.34%, substantially surpassing traditional approaches [18]. Li et al. subsequently developed an encoder and decoder architecture, Ind-CGRU, which integrates convolutional layers with gated recurrent units to capture long-range dependencies in overlapping messages. It likewise delivered strong performance in an experimental setting [1]. Although prior work has made progress, most existing ADS-B collision detection and separation methods rely on array signal processing or subspace decomposition techniques. These approaches assume that the receiver is equipped with an array of multiple antennas or operates under stable channel conditions. Therefore, they are not well suited to spaceborne receivers. To address these limitations, this paper targets collision scenarios involving two or three signals in single-channel satellite reception and develops two models: SplitNet-2, specialized for two-signal collisions, and SplitNet-3, designed for three-signal collisions.

The remainder of this paper is organized as follows. Section 2 describes typical ADS-B collision scenarios. Section 3 proposes signal separation models for collisions involving two signals and three signals. Section 4 provides a systematic experimental evaluation and analysis. Section 5 concludes the paper.

## 2. Signal Model

**Figure 1:**
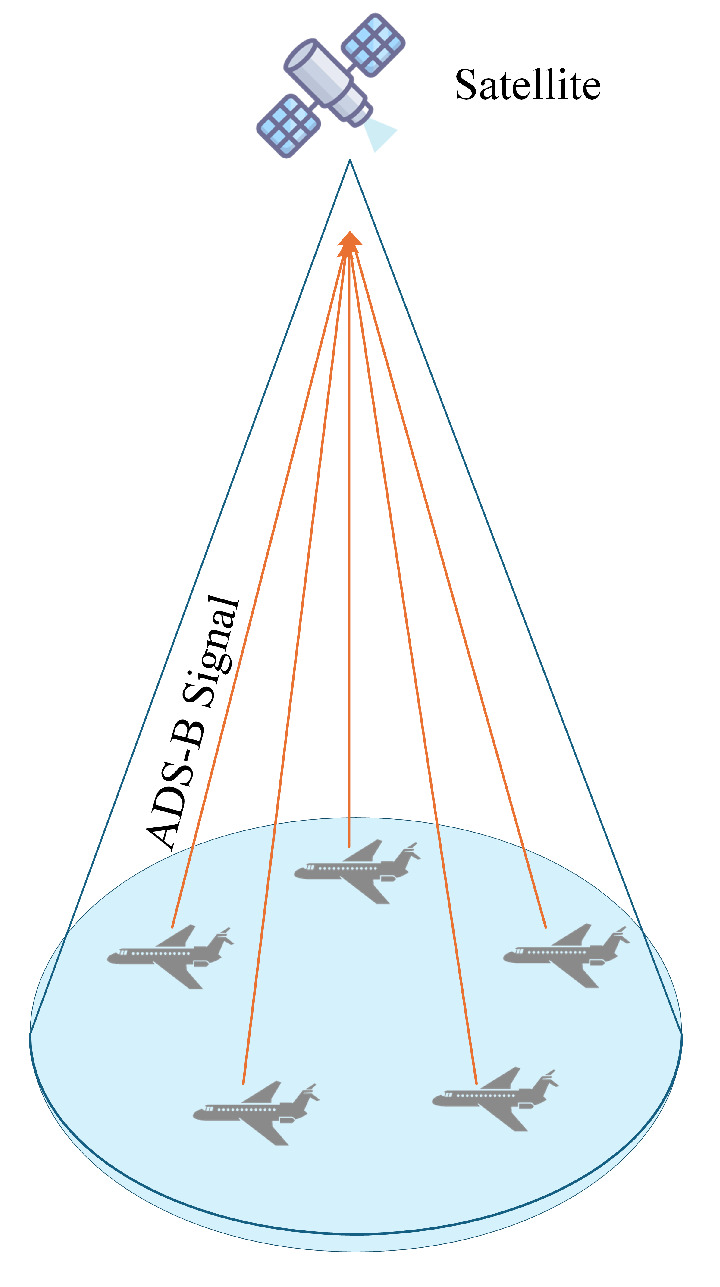
Space-based ADS-B signal collision scenario.

In a space-based ADS-B reception scenario, multiple aircraft within a single satellite beam may transmit messages nearly simultaneously. Because the beam footprint spans a wide area, each aircraft has a different slant range and radial velocity relative to the satellite. Consequently, the messages arrive with different propagation delays, Doppler shifts, and received powers. When two or more messages overlap in time within the receiver bandwidth, the signal received by the satellite is a composite waveform, the superposition of multiple transmissions. Figure 1 illustrates a typical space-based ADS-B scenario in which multiple aircraft within the same satellite beam transmit messages asynchronously.

In the physical layer, each 1090ES frame lasts 120 µs and consists of an 8 µs preamble and a 112 µs data block [19,20]. Under the 1090ES ADS-B broadcast mechanism, aircraft transmit independently. Therefore, message arrivals at the receiver can be approximated by a Poisson process with rate λ. Within a frame window Δt=120μs, the probability of observing a *k*-way overlap is

(1) $$P_{k}=\frac{{\left(\lambda \;\Delta t\right)}^{k}}{k!}\;e^{-\lambda \Delta t}.$$

**Figure 2:**
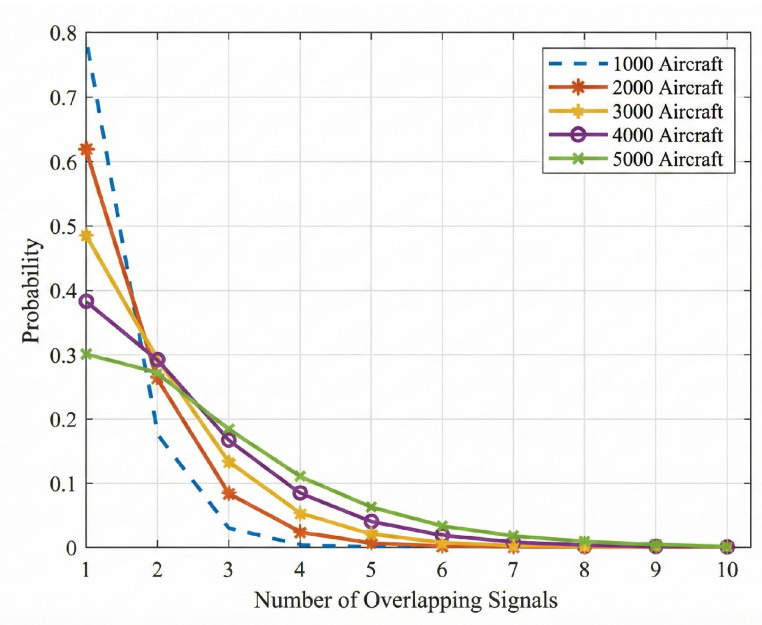
Space-based ADS-B signal collision probability.

Because $P_{k}$ decays factorially with *k*, two-signal and three-signal collisions dominate, and this work therefore focuses on separation under these two collision multiplicities. This trend is further confirmed by the collision multiplicity statistics in Figure 2 under varying aircraft density, where two-signal and three-signal collisions account for the majority of overlap events and higher multiplicities occur with much lower probability. In practice, the collision multiplicity is not assumed to be known a priori, and a lightweight front end is executed before separation to detect collisions and estimate whether the received mixture corresponds to a two- or three-signal collision. The estimated multiplicity is then used to select the corresponding separation network.

**Figure 3:**
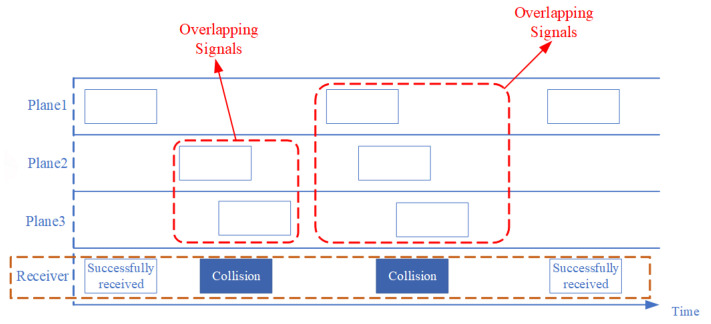
Illustration of time-domain overlaps.

Figure 3 shows the time-domain signals from multiple aircraft as received by the satellite. The horizontal axis denotes time, and dashed lines indicate the trajectories of individual messages. The blue frames at both ends are non-overlapping messages that fulfill ideal transmission conditions and can be fully decoded by the receiver. The frames marked by red dashed boxes correspond, respectively, to two- and three-signal collisions. Because the propagation delay is uncertain, multiple signals overlap in the time domain, causing severe distortion and structural corruption of the preamble and data block. This poses a severe decoding challenge, i.e., when multiple messages collide in time, a single-antenna receiver faces an underdetermined mixture of waveforms. Conventional decoding relies on reliable preamble detection and consistent bit timing, conditions that are difficult to maintain during collisions. Therefore, accurate modeling of propagation delays is essential, as the idealized assumption of perfectly aligned signals does not hold in practice. These challenges indicate that unknown timing offsets must be explicitly accounted for during signal separation.

On the basis of the above principle, we model both two-signal and three-signal collision scenarios. For the two-signal overlap scenario, the received waveform can be modeled as a superposition of two transmitted signals plus noise. To capture the realistic misalignment between arrivals, the received signal is expressed with explicit time offsets as follows:

(2) $$\begin{array}{c} x\left(t\right)=s_{1}\;\left(t-\tau_{1}\right)+s_{2}\;\left(t-\tau_{2}\right)+n\left(t\right), \end{array}$$

where $s_{1}\left(t\right)$ and $s_{2}\left(t\right)$ are the two transmitted ADS-B signals, while $\tau_{1}$ and $\tau_{2}$ are their unknown delays relative to the receiver clock. The three signal collision scenario, the received waveform extends to be a combination of three ADS-B signals with distinct delays, described as follows:

(3) $$x\left(t\right)=s_{1}\left(t-\tau_{1}\right)+s_{2}\left(t-\tau_{2}\right)+s_{3}\left(t-\tau_{3}\right)+n\left(t\right).$$

However, conventional separation algorithms that are based on such models exhibit limited performance under complex asynchronous conditions. Thus, we propose a signal separation framework based on deep learning and develop differentiated approaches for collision scenarios with varying numbers of overlapping signals. In particular, for the two-signal collision case, we design SplitNet-2, a single-head self-attention model that captures global dependencies across the entire time domain and achieves symmetric separation of the two signal sequences. For the more complex three-signal collision scenario, we propose SplitNet-3, which fuses one-dimensional convolution with residual connections. The model extracts fine-grained features from short temporal windows while integrating global contextual information.

## 3. Separation Method

In this section, we introduce SplitNet-2 and SplitNet-3 in detail.

### 3.1. SplitNet-2

**Figure 4:**
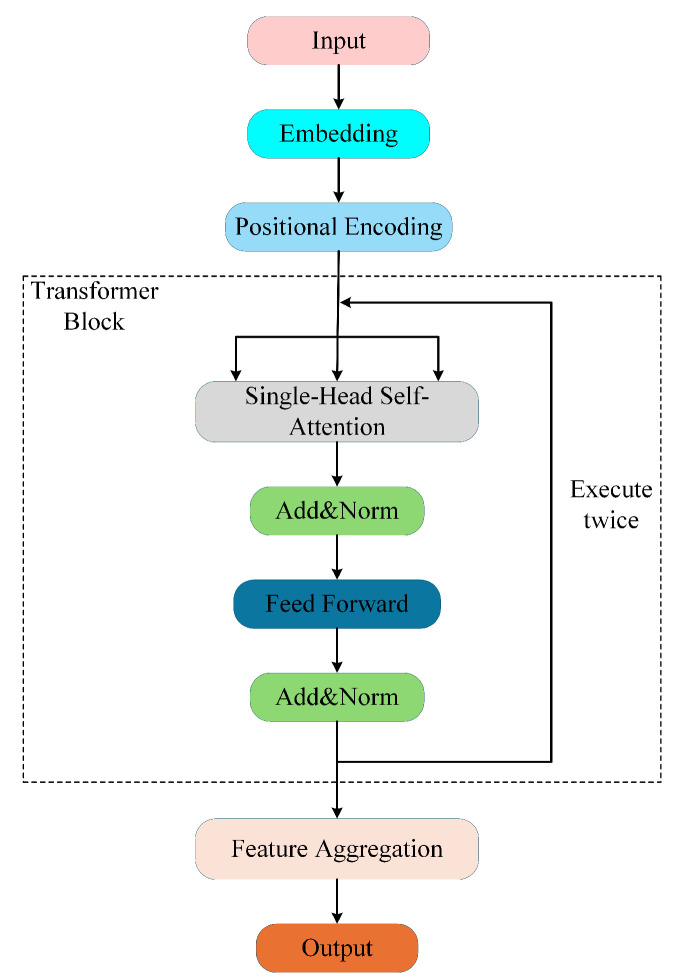
SplitNet-2 structure.

SplitNet-2 adopts a Transformer encoder architecture and models the separation of ADS-B signals with collisions involving two signals as a symmetric sequence labeling task [21]. The model performs a separate forward pass on each of the two input sequences and produces the corresponding separation prediction for each sequence independently. The overall network architecture is shown in Figure 4. The inputs are a collision signal sequence x(t) and its time-reversed sequence $\tilde{x}\left(t\right)=x\left(T-t\right)$. These inputs pass through an Embedding layer, a Positional Encoding layer, a Transformer block, and a Feature Aggregation layer, after which they are mapped to two output sequences of the same length as the input sequences, corresponding to the two separated source signals. The following subsections detail each component of the architecture using mathematical equations.

#### 3.1.1. Embedding

The input ADS-B collision signal is represented as a time series. To enhance feature representation, the model first maps the raw inputs into a high-dimensional feature space via the Embedding layer. Specifically, let the input signal sequence x(t) be of length *N*, with $C_{\mathrm{in}}$ channels per time sample. For a two-signal collision, the magnitude input has $C_{\mathrm{in}}=1$. We project each sample to a *d*-dimensional vector:

(4) $$h^{\left(0\right)}\left(n\right)=W_{e}\;x\left(n\right)+b_{e},$$

where $h^{\left(0\right)}\left(n\right)\in R^{d}$, $W_{e}\in R^{d\times C_{\mathrm{in}}}$, and $b_{e}\in R^{d}$ are learnable. From Equation (4), the model can obtain an initial feature sequence of length *N* and feature dimension *d*.

#### 3.1.2. Positional Encoding

Because the Transformer architecture does not explicitly encode token positions, positional information must be injected into the input sequence. We adopt the standard sinusoidal positional encoding and add a fixed positional vector to the token embeddings. Let $p\left(n\right)\in R^{d_{\mathrm{model}}\times 1}$ denote the positional encoding vector at position index *n* (starting from 0), where $d_{\mathrm{model}}$ is the embedding dimension. Its 2i and 2i+1 entries are given by the following:   

(5) $$\left\{\begin{array}{cc} p_{2i}\left(n\right) & =\sin\;\left(\frac{n}{{10,000}^{\frac{2i}{d_{\mathrm{model}}}}}\right),\\ p_{2i+1}\left(n\right) & =\cos\;\left(\frac{n}{{10,000}^{\frac{2i}{d_{\mathrm{model}}}}}\right), \end{array}\right.$$

where $i=0,\ldots ,\left\lfloor \frac{d_{\mathrm{model}}}{2}\right\rfloor -1$. The output of the Positional Encoding layer is as follows:

(6) $${\tilde{h}}^{\left(0\right)}\left(n\right)=h^{\left(0\right)}\left(n\right)+p\left(n\right).$$

#### 3.1.3. Transformer Block

SplitNet-2 consists of two cascaded Transformer blocks. Each block first performs single-head self-attention and then applies a feedforward neural network. After each computation, the model adds a residual connection and applies layer normalization.

In a Transformer, self-attention realizes information exchange via the Query–Key–Value mechanism. For the input feature sequence of the current layer, $\left(h^{\left(l-1\right)}\left(1\right),\;\ldots ,\;h^{\left(l-1\right)}\left(N\right)\right)$, we compute the corresponding query, key, and value vectors $\left(q^{\left(l\right)},\;k^{\left(l\right)},\;v^{\left(l\right)}\right)$ by linear projections:   

(7) $$\left(q^{\left(l\right)}\left(n\right),\;k^{\left(l\right)}\left(n\right),\;v^{\left(l\right)}\left(n\right)\right)=\left[W_{Q}^{\left(l\right)},\;W_{K}^{\left(l\right)},\;W_{V}^{\left(l\right)}\right]\;h^{\left(l-1\right)}\left(n\right),$$

where $W_{Q}^{\left(l\right)},\;W_{K}^{\left(l\right)},\;W_{V}^{\left(l\right)}\in R^{d\times d}$ and l=1,2.

For any two positions *n* and *m* in the sequence, we define their attention score by the dot product between the query at *n* and the key at *m*. After scaling and normalization, the attention weight is

(8) $$\alpha_{n,m}^{\left(l\right)}=\frac{\exp\;\left(q^{\left(l\right)}{\left(n\right)}^{\;⊤}k^{\left(l\right)}\left(m\right)/\sqrt{d}\right)}{\sum_{u=1}^{N}\exp\;\left(q^{\left(l\right)}{\left(n\right)}^{\;⊤}k^{\left(l\right)}\left(u\right)/\sqrt{d}\right)}.$$

The output vector at position *n* is given by the attention-weighted sum of the value vectors:   

(9) $$o^{\left(l\right)}\left(n\right)=\sum_{m=1}^{N}\alpha_{n,m}^{\left(l\right)}\;v^{\left(l\right)}\left(m\right).$$

Then, a residual connection with layer normalization is applied to ensure numerical stability and mitigate vanishing gradients:

(10) $${\tilde{h}}^{\left(l\right)}\left(n\right)=\mathrm{LayerNorm}\left(h^{\left(l-1\right)}\left(n\right)+o^{\left(l\right)}\left(n\right)\right).$$

After obtaining the self-attention output, each Transformer block proceeds to a feedforward neural network (FFN) that applies a nonlinear transformation independently at each time position to further extract position-independent nonlinear features. The computation is given by the following:

(11) $$\left\{\begin{array}{c} u^{\left(l\right)}\left(n\right)=W_{1}^{\left(l\right)}\;{\tilde{h}}^{\left(l\right)}\left(n\right)+b_{1}^{\left(l\right)},\\ z^{\left(l\right)}\left(n\right)=\mathrm{ReLU}\;\left(u^{\left(l\right)}\left(n\right)\right),\\ h^{\left(l\right)}\left(n\right)=W_{2}^{\left(l\right)}\;z^{\left(l\right)}\left(n\right)+b_{2}^{\left(l\right)} \end{array}\right.$$

where $W_{1}^{\left(l\right)}\in R^{d_{\mathrm{ff}}\times d}$, $W_{2}^{\left(l\right)}\in R^{d\times d_{\mathrm{ff}}}$, and another residual–norm pair finalizes the layer:

(12) $$h^{\left(l\right)}\left(n\right)\leftarrow \mathrm{LayerNorm}\left({\tilde{h}}^{\left(l\right)}\left(n\right)+h^{\left(l\right)}\left(n\right)\right).$$

Two such Transformer layers (l=1,2) are stacked, giving ${\left(h^{\left(2\right)}\left(n\right)\right)}_{n=1}^{N}$.

#### 3.1.4. Feature Aggregation

For each time position *n*, the feature vector $h^{\left(2\right)}\left(n\right)$ is fed into a fully connected layer to produce logits z(n), which are then passed through a sigmoid function to obtain a presence probability:

(13) $$z\left(n\right)=W_{o}\;h^{\left(2\right)}\left(n\right)+b_{o},\;\hat{y}\left(n\right)=\sigma \left(z\left(n\right)\right),\;\hat{y}\left(n\right)\in {\left(0,1\right)}^{2}.$$

With $W_{o}\in R^{2\times d}$ and $b_{o}\in R^{2}$, the two channels of $\hat{y}\left(n\right)$ represent the probabilities that the sample at time index *n* belongs to source 1 and source 2, respectively. During training, the loss is computed on logits z(n), while $\hat{y}\left(n\right)$ provides a probabilistic interpretation for inference and visualization. Running the same pipeline on $\tilde{x}\left(t\right)$ produces an independent probability pair, and the two outputs together constitute the separated signals.

### 3.2. SplitNet-3

**Figure 5:**
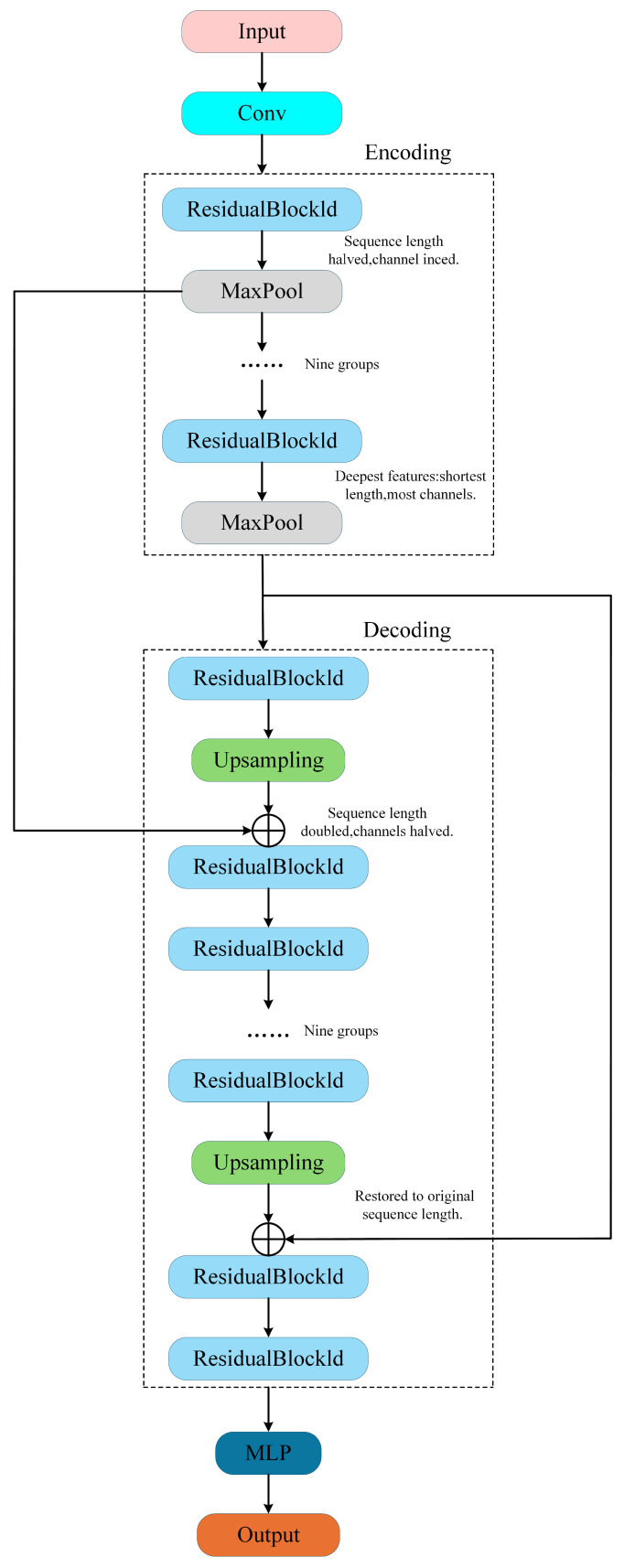
SplitNet-3 structure.

SplitNet-3 adopts a one-dimensional U-Net encoder–decoder architecture [22]. On the basis of a symmetric structure, it replaces the two-dimensional convolutions used in image segmentation with one-dimensional convolutions for time series and introduces residual connections at each scale to alleviate vanishing gradients in deep networks and improve training stability. Unlike SplitNet-2, SplitNet-3 takes only the raw mixed waveform x(t) as input and uses three parallel output branches, each predicting the waveform of one demixed signal. The model architecture is shown in Figure 5. The encoder part performs sequence compression and hierarchical feature extraction, the decoder part performs upsampling and signal reconstruction, and the skip connections transmit features across layers to enhance information synchronization between the encoder and decoder.

#### 3.2.1. Encoder

Let $H_{0}\left(t\right)=x\left(t\right)$ denote the input waveform. Prior to the encoder, a one-dimensional convolutional layer transforms $H_{0}$ into an intermediate feature map. The conversion formula is as follows:

$$z_{1}\left(t\right)=f\left(W_{1}*H_{0}\left(t\right)+b_{1}\right)$$

where $W_{1}$ and $b_{1}$ are the convolutional kernel and bias for stage 1, ∗ denotes convolution, and f(·) is an elementwise nonlinear activation.

The encoder then consists of multiple sequential downsampling stages that progressively reduce the temporal resolution of the input while extracting higher-level feature representations. Furthermore, to capture deeper features, each encoder stage includes a residual block (described below); thus, the stage output equals the sum of its input features and the block’s transform.

Let ${\mathrm{ResBlock}}_{i}\left(\;\cdot \;\right)$ represent the residual block at encoder stage *i*. Then the output of encoder stage *i* (after the residual block) can be written as follows:

(14) $$E_{i}\left(t\right)={\mathrm{ResBlock}}_{i}\left(H_{i-1}\left(t\right)\right)=H_{i-1}\left(t\right)+g_{i}\left(H_{i-1}\left(t\right)\right),$$

where $g_{i}\left(\;\cdot \;\right)$ denotes the composition of convolution layers and nonlinearities within the *i*th residual block, while $H_{i-1}\left(t\right)$ is the input to that block (with $H_{0}\left(t\right)=x\left(t\right)$). The residual connection $H_{i-1}\left(t\right)+g_{i}\left(H_{i-1}\left(t\right)\right)$ helps preserve information from earlier layers. After each residual block, a downsampling operation is applied to reduce the temporal length. For example, with pooling or a strided convolution of factor 2, the downsampled output $H_{i}\left(t\right)$ retains every second time step of $E_{i}\left(t\right)$:

(15) $$H_{i}\left(t\right)=E_{i}\left(2\;\cdot \;t\right).$$

This downsampled feature map $H_{i}\left(t\right)$ serves as the input to the next encoder stage. Stacking *L* encoder stages yields a bottleneck representation $H_{L}\left(t\right)$ at the lowest temporal resolution, capturing the broad temporal context of the input.

#### 3.2.2. Decoder

The decoder mirrors the encoder through a sequence of upsampling stages that gradually restore temporal resolution while combining encoder features through skip connections. Let $Z_{L}\left(t\right)=H_{L}\left(t\right)$ denote the bottleneck feature map. The decoder begins by upsampling this lowest-resolution feature map:

(16) $$Z_{L}^{↑}\left(t\right)=\mathrm{UpSample}\left(Z_{L}\left(t\right)\right),$$

where UpSample increases the length of $Z_{L}$ by a factor *s*, which is the inverse of the encoder’s downsampling factor. The upsampled output $Z_{L}^{↑}\left(t\right)$ is then concatenated with the encoder feature at the same scale through a skip connection:

(17) $$U_{L-1}\left(t\right)=\mathrm{Concat}\left(Z_{L}^{↑}\left(t\right),\;E_{L-1}\left(t\right)\right),$$

This fused representation $U_{L-1}\left(t\right)$ is then fed into a residual block in the decoder stage to further process and refine the features. For decoder stage *i*, we similarly have

(18) $$Z_{i}\left(t\right)={\mathrm{ResBlock}}_{i}^{\left(dec\right)}\left(U_{i}\left(t\right)\right)=U_{i}\left(t\right)+h_{i}\left(U_{i}\left(t\right)\right),$$

where ${\mathrm{ResBlock}}_{i}^{\left(dec\right)}$ denotes the residual block at decoder stage *i*, while $h_{i}\left(\;\cdot \;\right)$ denotes the internal convolutional transformations at that stage. After the residual block, the decoder continues processing at the next resolution up; $Z_{i}\left(t\right)$ is upsampled and concatenated with the encoder output $E_{i-1}\left(t\right)$ from the adjacent higher-resolution encoder stage. This process repeats until the highest resolution corresponding to the original signal length is reached.

#### 3.2.3. MLP

SplitNet-3 does not adopt the common single-layer convolution direct mapping head but instead employs a three-layer MLP composed of fully connected layers as the output head. This module generates three independent output sequences in parallel, corresponding, respectively, to the three ADS-B signals. Let $Z_{0}\left(t\right)$ denote the high-resolution feature map output by the final decoder residual block. The output head is a 1×1 pointwise convolution that maps $Z_{0}\left(t\right)$ to three channels, followed by a sigmoid mapping ${\hat{y}}_{j}\left(t\right)=\sigma \;\left(z_{j}\left(t\right)\right)$ to obtain probability-like outputs for interpretation and inference.

(19) $$z_{j}\left(t\right)=W_{\mathrm{out},j}*Z_{0}\left(t\right)+b_{\mathrm{out},j},\;{\hat{y}}_{j}\left(t\right)=\sigma \;\left(z_{j}\left(t\right)\right),\;\;j=1,2,3.$$

In our implementation, the loss is evaluated on logits $z_{j}\left(t\right)$, and ${\hat{y}}_{j}\left(t\right)$ is reported only as a probabilistic interpretation of the predicted bits.

## 4. Training and Results

### 4.1. Loss Function Design

In this study, for each output channel, the signal separation task is formulated as a binary classification problem. Accordingly, we train the network using the Binary Cross-Entropy with Logits Loss (BCE with Logits Loss). The loss function is defined as

(20) Loss(y,z)=−(y·log(σ(z))+(1−y)·log(1−σ(z))),

where *z* denotes the raw model outputs and y∈{0,1} represents the ground-truth binary. For the SplitNet-2 model, assume the two output signal predictions are ${\hat{s}}_{1}$ and ${\hat{s}}_{2}$ with corresponding ground truths $s_{1}$ and $s_{2}$. The individual losses are computed as follows:

(21) $${\mathrm{Loss}}_{i}=-\left(s_{i}\;\cdot \;\log\left(\sigma \left(z_{i}\right)\right)+\left(1-s_{i}\right)\;\cdot \;\log\left(1-\sigma \left(z_{i}\right)\right)\right),$$

where $z_{i}$ denotes the logit predicted by the network and $s_{i}$ the ground-truth label at the corresponding time sample, with i∈{1,2}.

The model’s total training loss is defined as

(22) $${\mathrm{Loss}}_{\mathrm{total}}=\frac{{\mathrm{Loss}}_{1}+{\mathrm{Loss}}_{2}}{2}.$$

The SplitNet-3 training procedure follows the same supervised learning framework as that of SplitNet-2 and continues to use pointwise BCE as the primary loss function. For the *j*th output channel with j∈{1,2,3}, let $z_{j}$ denote the predicted logit (pre-sigmoid) and let $s^{\left(j\right)}\in \left\{0,1\right\}$ denote the corresponding ground-truth label. The probability-like output is ${\hat{s}}^{\left(j\right)}=\sigma \;\left(z_{j}\right)$, which is used for interpretation and inference. The loss is defined as:

(23) $${\mathrm{Loss}}_{j}=-\left(s^{\left(j\right)}\log\left(\sigma \left(z_{j}\right)\right)+\left(1-s^{\left(j\right)}\right)\log\left(1-\sigma \left(z_{j}\right)\right)\right),\;j\in \left\{1,2,3\right\}$$

The overall loss is defined as

(24) $${\mathrm{Loss}}_{\mathrm{total}}=\frac{{\mathrm{Loss}}_{1}+{\mathrm{Loss}}_{2}+{\mathrm{Loss}}_{3}}{3}.$$

This loss function is designed to ensure that the network assigns equal priority to the reconstruction quality of all input signals and to prevent the model from favoring the reconstruction of any single signal while neglecting the others. Additionally, this approach significantly improves overall decoding performance and accelerates training convergence, and it is particularly effective at preventing early training phases in which only a single signal is recovered while the others are ignored.

### 4.2. Preparation of Training Data

For the preparation of training data, we use a data generator to synthesize simulated colliding signal samples spanning diverse power ratios and delay combinations, thereby improving the model’s adaptability to real-world channel conditions. As we focus on separating two- and three-signal ADS-B collisions, all samples in the dataset are generated by superimposing two or three ADS-B signals under varying channel conditions. Taking practical system constraints into account, we set the sample parameter ranges as follows: relative delay 0–120 μs, inter-signal power difference 0–5 dB, and SNR 5–20 dB.

In this experiment, 10 samples are taken per microsecond, corresponding to a sampling rate of 10 MHz. A single ADS-B 1090ES frame has a duration of 120 microseconds, yielding 1200 time-domain samples. Binary labels are constructed with a chip resolution of 0.5 microseconds, resulting in a total label length of 240. The noise model is additive white Gaussian noise. Two independent datasets are generated for two-source and three-source collision scenarios to train SplitNet-2 and SplitNet-3, respectively. The two-source dataset contains 1400 training samples and 600 validation samples, while the three-source dataset contains 7000 training samples and 3000 validation samples. The reported results are obtained from 20,000 Monte Carlo trials. The pseudo-code is given in Algorithm 1.
**Algorithm 1** Dataset generation for SplitNet-*M***Input:** M∈{2,3}, message length $L_{\mathrm{msg}}$, buffer length *L*, SNR ranges ${\left\{S_{i}\right\}}_{i=1}^{M}$**Output:** Mixture *x*, labels *Y*1:**for** i=1**to***M***do**2:   Generate message waveform $s_{i}$ and label $b_{i}$, and draw start index $t_{i}$.3:**end for**4:x←0.5:**for** i=1**to***M***do**6:   Draw ${\mathrm{SNR}}_{i}∼S_{i}$ and set $p_{i}\leftarrow 10^{{\mathrm{SNR}}_{i}/20}$.7:   $x\left[t_{i}:t_{i}+L_{\mathrm{msg}}-1\right]\leftarrow x\left[t_{i}:t_{i}+L_{\mathrm{msg}}-1\right]+p_{i}s_{i}$.8:**end for**9:Add AWGN to *x*.10:$Y\leftarrow [b_{1};\;b_{2};\;\ldots ;\;b_{M}]$.11:**return** (x,Y).

SplitNet-2 is optimized using Adam with an initial learning rate of $1\times 10^{-4}$, a mini-batch size of 128, and a maximum of 1000 epochs. Model performance is evaluated on the validation set after each epoch. Early stopping is employed, with patience = 50 epochs, and the final model is selected as the checkpoint yielding the lowest validation loss.

SplitNet-3 is trained using AdamW to improve optimization stability for the more challenging three-source separation task. The initial learning rate is set to $1\times 10^{-3}$ and decays during training; when the validation metric reaches a plateau, the learning rate is further reduced to $1\times 10^{-4}$ and subsequently to $1\times 10^{-5}$ for fine-tuning. The mini-batch size is 64, and the maximum number of epochs is 1500. All weights are initialized using Xavier uniform initialization. To alleviate overfitting, a dropout layer with a rate of 0.1 is inserted after selected convolution blocks when needed. Early stopping and checkpoint selection follow the same criterion as SplitNet-2. The pseudo-code is given in Algorithm 2.
**Algorithm 2** Training pipeline for SplitNet models**Input:** Model $f_{\theta }$, generator D, batch size *B*, learning rate η, max epochs *E***Output:** Best checkpoint $\theta^{★}$1:Initialize the optimizer (AdamW) and loss function L (BCEWithLogitsLoss).2:Initialize the best criterion (e.g., best loss ←+∞).3:**for** e=1**to***E***do**4:  **for all** mini-batches $\left(x_{b},Y_{b}\right)$
**do**5:     ${\hat{Y}}_{b}\leftarrow f_{\theta }\left(x_{b}\right)$.6:     $\ell \leftarrow L({\hat{Y}}_{b},Y_{b})$.7:     Backpropagate and update θ.8:  **end for**9:  Update the learning rate, save a checkpoint if the criterion improves.10:**end for**11:**return** $\theta^{★}$.

### 4.3. Results of SplitNet-2

**Figure 6:**
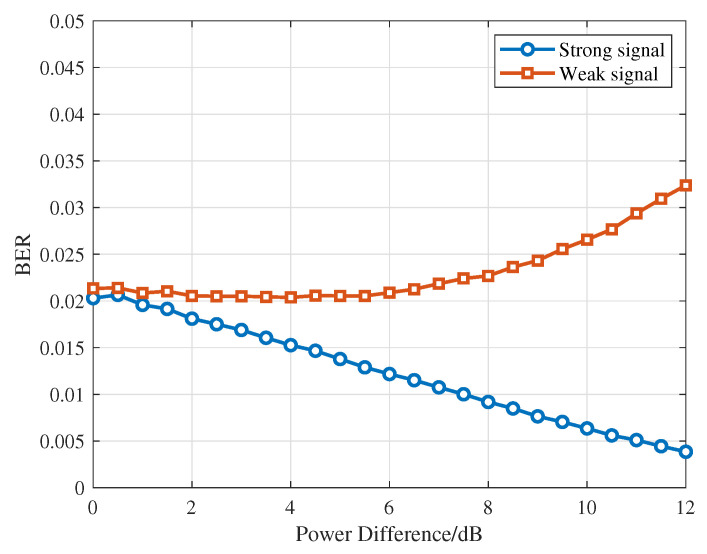
BER for different power differences.

To evaluate the signal separation performance of the SplitNet-2 model under varying conditions, we conducted tests with two source signals configured with a power difference of 0–5 dB and an SNR of 5–20 dB, using the bit error rate (BER) as the performance metric. Here, BER is defined as the fraction of incorrectly recovered bits with respect to the ground truth within a frame. It is obtained by performing a bitwise comparison between the separated output bit sequence and its corresponding reference sequence and is then averaged over all test frames under each SNR and power difference setting. Figure 6 illustrates the separation performance of the two signals under different power differences. The results indicate that both the stronger and weaker signals maintain low bit error rates across varying power differences, demonstrating that the model retains strong separation robustness even when one signal dominates. Meanwhile, the strong signal shows greater adaptability under large power differences.

To further comprehensively evaluate the effectiveness of the proposed method, the simulations incorporate the overlapping-signal processing approach recommended in the ICAO standard documents [20], along with two representative single-antenna signal separation algorithms as baselines, specifically including the following:

- CD: This method makes a determination based on either the received signal power or the order of arrival, retaining only the stronger or earlier-arriving signal while directly discarding the weaker or later-arriving one.
- TDBSS: By modeling the statistical characteristics of time-domain signals, this method extracts signals within a blind source separation framework without requiring prior information [15].
- PASA: This method employs a parametric adaptive projection model for dynamic estimation and reconstruction of overlapping signals [23].

**Figure 7:**
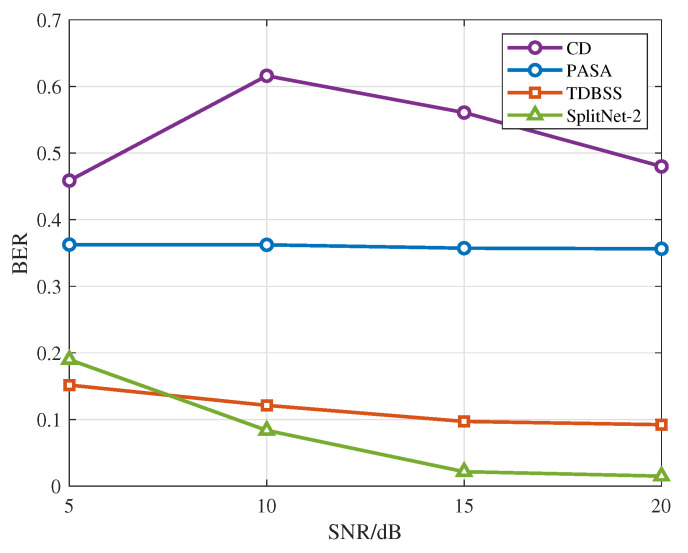
BER as a function of SNR for different algorithms.

As shown in Figure 7, CD exhibits the highest BER over the entire SNR range, whereas PASA is largely insensitive to SNR and maintains a BER close to 0.36 throughout. TDBSS achieves a substantially lower BER than CD or PASA, and its BER improves steadily as the SNR increases, reaching approximately 0.10 at 15 dB and 0.09 at 20 dB. The proposed SplitNet-2 demonstrates the strongest SNR-dependent gain, reducing the BER from about 0.19 at 5 dB to about 0.09 at 10 dB and further to less than 0.03 for SNR values of 15 dB and above. At 5 dB, SplitNet-2 is slightly inferior to TDBSS, which is consistent with the fact that TDBSS primarily leverages inter-signal power disparity and can remain effective in low-SNR conditions when the collision region and relative delay are accurately identified. However, TDBSS is highly sensitive to collision localization and delay alignment errors, and its performance can deteriorate sharply when collision detection is imperfect, particularly when the SNR is low [15]. In contrast, SplitNet-2 performs separation directly from the mixed waveform without a dedicated collision detection and localization stage; therefore, the proposed method achieves better overall performance than TDBSS.

### 4.4. Results of SplitNet-3

**Figure 8:**
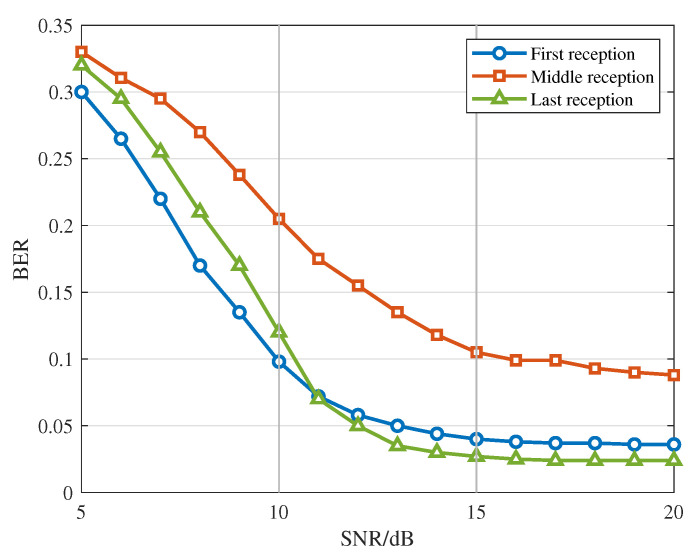
BER as a function of SNR for signals with different reception times.

For the three-signal collision scenario, we evaluate the separation performance of three overlapping ADS-B signals under varying SNRs at power differences of 0–3 dB, again using BER as the evaluation metric. Figure 8 shows the BER curves versus SNR for three signals with distinct arrival times. Because the signal arriving in the middle overlaps with both the first and third signals, its BER is noticeably higher than those of the other two.

To evaluate the effectiveness of the proposed SplitNet-3 model, we include two performance baselines in the simulations:

- Non-colliding signals that are decoded directly at the receiver, serving as a theoretical upper bound on performance.
- Mixed signals decoded by a conventional decoder when the arrival times of all three signals are known.

**Figure 9:**
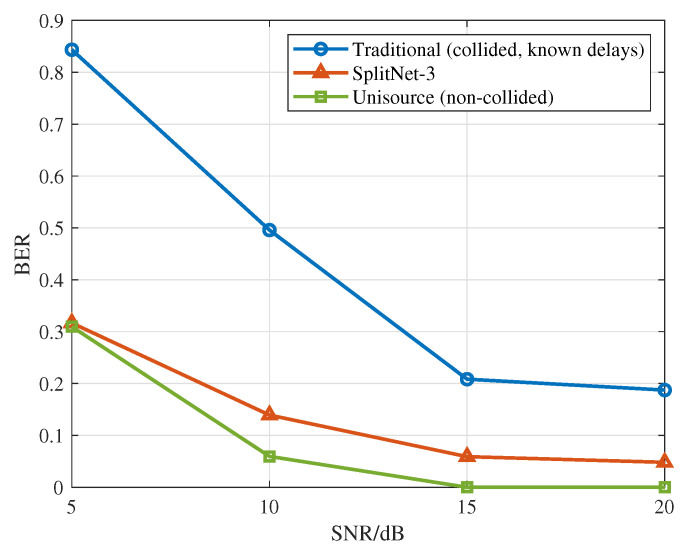
BER performance comparison under different SNRs.

Figure 9 presents the simulation results. The proposed SplitNet-3 maintains a low BER, indicating strong signal separation across a range of complex communication environments.

## 5. Conclusions

This paper has proposed two deep learning–based signal separation models specifically designed to address the separation of colliding ADS-B signals in single-channel satellite reception. SplitNet-2 is tailored to separate collisions involving two signals. It employs a self-attention mechanism with a single head and a symmetric architecture with two inputs and two outputs, enabling the modeling of dependencies between signals across the entire time sequence and achieving highly accurate sequence reconstruction. SplitNet-3 targets the more complex case of three superimposed signals. It uses a 1D U-Net backbone enhanced with multiple residual blocks to improve feature extraction and training stability, and its decoder introduces a global flattening layer and an MLP module that enable the model to capture both local pulse features and global context, thereby achieving parallel separation of the three signals. Simulation results have shown both models to achieve strong separation performance under conditions of high noise and strong interference. SplitNet-2 has achieved a BER below 0.1 in the scenario with two colliding signals. With the overlap of three signals, SplitNet-3 has maintained a low average BER and has achieved a separation success rate exceeding 90% at moderate to high SNR. Compared with conventional decision methods in the time domain based on energy or thresholds, the proposed models have delivered significant improvements in separation accuracy, robustness, and practical applicability, thereby demonstrating strong application potential and engineering value for spaceborne ADS-B systems. 

## Data Availability

The original contributions presented in this study are included in the article. Further inquiries can be directed to the corresponding author.
